# Abdominal wall recurrence of a gastrointestinal stromal tumor: case report

**DOI:** 10.1186/s40064-015-1220-3

**Published:** 2015-08-19

**Authors:** Hajar Hachim, Anass Mohammed Majbar, Mouna Alaoui, Mohamed Raiss, Farid Sabbah, Abdelmalek Hrora, Mohamed Ahallat

**Affiliations:** Clinique Chirurgicale C, Ibn Sina University Hospital, 10090 Rabat, Morocco

**Keywords:** Gastrointestinal stromal tumor, Laparoscopic surgery, Neoplasm recurrence

## Abstract

**Introduction:**

The gastrointestinal stromal tumors (GIST) are mesenchymal tumors, most commonly affecting the stomach and small bowel. Only few cases of port-site recurrence after laparoscopic treatment have been reported. We herein report the case of a parietal recurrence on the extraction incision site, 7 years after laparoscopic surgery for small bowel GIST.

**Case report:**

A 47 years-old female patient was hospitalized in November 2007 for isolated pelvic pain. CT scan showed an intestinal tumor with a benign aspect measuring 50 mm. A laparoscopy-assisted resection was performed. Surgical exploration found a 7 cm small bowel tumor. It was extracted through a supra-pubic transversal incision without a wound protector and then resected. Histologic analysis revealed an intestinal GIST with high aggressive potential (five mitosis per field), with CD117 positive at the immunohistochemical examination. The patient had no adjuvant chemotherapy. Seven years later, the patient was readmitted for an abdominal mass at the site of the supra-pubic scar. Abdomino-pelvic CT scan showed a 10 × 7.5 cm solid mass of the abdominal wall. Percutaneous biopsies were done and the pathological analysis revealed a mesenchymal-cell tumor, positive to CD117 and DOG1 at the immunohistochemical examination. Final diagnosis was abdominal wall recurrence of GIST secondary to tumor-contamination during the first surgery.

**Conclusion:**

Abdominal wall recurrence of GIST after laparoscopic surgery is rarely reported. This complication should be avoided with preventive measures such as the use of extraction bags or wound protectors.

## Introduction

Gastrointestinal stromal tumors are rare tumors secondary to a malignant proliferation of mesenchymal cells. They affect more frequently the stomach and the small bowel in comparison with other segments of the digestive tract (Landi et al. 2015).

Enbloc surgical resection (R0) with a safety margin of 1–2 cm is actually the only potentially curative treatment. Imatinib based adjuvant chemotherapy is complementary to the surgery to improve the survival and the disease free survival. Laparoscopy has been used successfully in the treatment of digestive GIST with minor complications. Particularly, very few cases of port sites recurrences have been reported (Furukawa et al. 2012).

We herein report the case of an abdominal wall recurrence on the site of the extraction incision of a laparoscopically resected small bowel stromal tumor.

## Case report

**Fig. 1:**
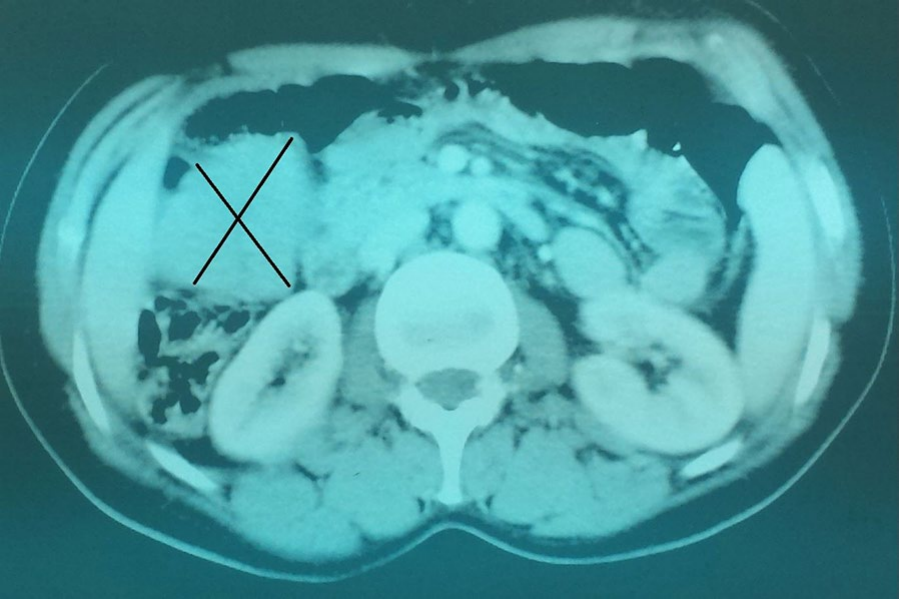
Computed tomography showing the intestinal tumor

We report the case of a 47 years-old female patient, who was referred to our unit in November 2007 for isolated pelvic pain. Clinical examination was without abnormalities. The abdominal pelvic ultrasound revealed a 60 mm hypoechoic and heterogeneous mass in the right iliac fossa. The abdominal pelvic computed tomography showed a tumor measuring 50 × 50 × 48 mm, attached to the anterior wall of the cecum and the right colon (Fig. 1).

The multidisciplinary meeting decided to perform a diagnostic and therapeutic laparoscopy. The laparoscopic exploration showed a 70 mm tumor of the small bowel, 70 cm upstream the last ileal loop. The tumor was extracted through a small suprapubic incision, without the use of a wound protector. It was resected with a safety margin of 2 cm and an end-to-end anastomosis was performed. The postoperative course was marked by an infection of the extraction’s site. The pathology specimen findings revealed a stromal tumor with a high aggressive potential (five mitosis per field), positive to CD117 at the immunohistochemical examination. An Imatinib adjuvant therapy was indicated, but the patient did not receive it because of financial considerations.

The patient had a postoperative follow-up (clinical examination, abdominal ultrasonography and abdominal computed tomography alternately) every 3 months during the first year, then every 6 months during the second and the third year. After that, the patient stopped her follow-up on her own.

**Fig. 2:**
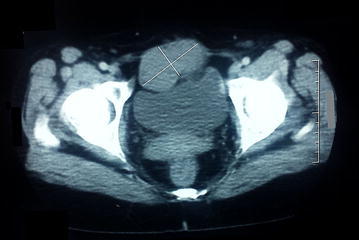
Computed tomography showing the abdominal wall recurrence

Seven years after the surgical resection, the patient was hospitalized for a pelvic mass underneath the old scar. The abdominal examination found a mass measuring 60/70 mm. The abdominal pelvic CT scan showed a 100/75 mm solid mass of the abdominal wall alongside to the pubic symphysis (Fig. 2).

Percutaneous biopsies of the tumor were made and histologic examination showed an aspect of low-grade malignancy GIST. An enbloc surgical resection of the tumor was performed, with a safety margin of 1 cm. The wall defect measured 6 cm in diameter and it was closed by direct suture. The pathological examination revealed a tumoral proliferation of spindle atypical cells with mitotic signs. The immunehistochemical examination showed an important positivity to CD117 and DOG1. The patient received adjuvant chemotherapy (Imatinib).

## Discussion

We herein report the case of parietal recurrence on the extraction site of a GIST, after a laparoscopy-assisted resection. This is a rare event and only few cases have been reported in the literature, mainly port-site metastasis (Kaczmarek et al. 2001; Davies et al. 2008; Furukawa et al. 2012) (Table 1).

**Table 1:** Summary of previously reported cases of GIST parietal recurrence in the literature (Kaczmarek et al. 2001; Davies et al. 2008; Furukawa et al. 2012)

	Localization	Tumor size	Intervention	Recurrence site	Specimen results/mitotic index	Extraction preventive measures
Kaczmarek et al. (2001)	Stomach GIST	4 × 4 cm	Endoscopic wedge resection	Port-site metastasis	>5	No information
Davies et al. (2008)	Stomach GIST	13 cm	Laparoscopic diagnostic + biopsies	Port-site metastasis	Malignant GIST+ Strongly positive CD117	No information
Furukawa et al. (2012)	Stomach GIST	–	Laparoscopic partial gastrectomy	Umbilical wound recurrence	GIST c-Kit+	No information

Several mechanisms may be responsible of GIST parietal recurrences (on the port sites of the extraction incisions) (Martinez et al. 1995; Schaeff et al. 1998) including: the high pressure of CO_2_ insufflation; the excessive manipulation of the tumor during surgery (stromal tumors are easily friable because of their necrotic character) (Landi et al. 2015), and secondary dissemination because of lack of protection of the abdominal wall during specimen extraction.

This complication is preventable with simple measures such as: the extraction of the specimen in a wound protector, careful manipulation of the tumor to avoid breaking; peritoneal washing with cytocidal or anti-adhesive solution (Kaczmarek et al. 2001; Shoup et al. 2002; Davies et al. 2008; Furukawa et al. 2012) and even the resection of the port sites. The use of the wound protector aims to protect the wound edges from contamination (infectious or tumoral). A meta-analysis of randomized controlled trials evaluating the use of wound protectors in gastrointestinal and biliary surgical procedures and the impact on surgical site infections (SSIs) showed nearly 50 % decrease in SSIs (RR = 0.55; 95 % CI, 0.31–0.98; p = 0.04) just by using a wound protector (Edwards et al. 2012). In our case, not using a wound protector was responsible of a SSI immediately after the surgery and a tumor contamination responsible of a parietal recurrence 7 years later.

The influence of the surgeon was also suggested responsible of 0–21 % of port-site recurrences after a laparoscopy performed for cancer. An experimental study on a porcine model showed a tumor recurrence in 63.8 % in the control group versus 13.8 % in the protective measures group (Schneider et al. 2001) suggesting that the use of standardized protective measures might reduce greatly the incidence of port-site recurrence (Franklin et al. 1996). The protective measure in this study were: prevention of gas leaks, fixation of trocars, protection of the extraction port, rinsing of instruments with cytotoxic substances such as povidone iodine or hydrogen peroxyde, and closure of peritoneal wounds (Wang et al. 1999).

Although our patient had a primary resectable localized GIST, a complete resection with negative microscopic margins do not prevent recurrences. The estimation of recurrence and death risks related to the GIST is based on the tumor size, mitotic rate and the site of the tumor. This estimation allows the selection of patients for adjuvant Imatinib and identifying their prognosis (Fletcher et al. 2002; Miettinen and Lasota 2006; Dematteo et al. 2008) (Tables 2, 3). Our patient had initially a 70 mm tumor, located in the small bowel, with five mitosis per field, positive to CD117 and no protection measures during the extraction procedure. So it would be reasonable to label her into a “high risk” category, even though she had an intermediate risk of recurrence (24 %) according to the stratification models (Fletcher et al. 2002; Miettinen and Lasota 2006). A study comparing 3 vs 1 year of Imatinib (400 mg/day) in the high-risk GIST patients showed that the recurrence-free survival was 86.6 % in 3-years arm versus 60.1 % in the 1-year arm. Five-years overall survival was also significantly better in the 3-years Imatinib arm versus the 1-year arm (92 vs. 81.7 %, p = 0.019) (George et al. 2009). Our patient did not receive Imatinib after the first surgery. Adjuvant chemotherapy would have reduced the risk of recurrence (Wang et al. 1999).

**Table 2:** Estimation of the recurrence risk or death linked to the disease in localized and resecable GISTs depending on tumor size and the mitotic index (Fletcher et al. 2002)

Risk	Maximum diameter	Mitotic index^a^
Very low risk	<2 cm	<5
Low risk	2–5 cm	<5
Intermediate risk	<5 cm 5–10 cm	6–10 <5
High risk	>5 cm >10 cm “Whatever”	>5 “Whatever” >10

^a^Per 50 fields

**Table 3:** Estimation of the recurrence risk or death linked to the disease in localized and resecable GISTs depending on tumor size, the tumor localization and the mitotic index (Miettinen and Lasota 2006)

Tumor’s maximum diameter (cm)	Mitotic index^b^	Stomach GIST (%)	Small bowel GIST (%)	Duodenal GIST (%)	Rectal GIST (%)
≤2	≤5	0	0	0	0
>2 ≤ 5	≤5	1.9	4.3	8.3	8.5
>5 ≤ 10	≤5	3.6	24	–^a^	–^a^
>10	≤5	12	52	34	57
≤2	>5	0	50	–^a^	54
>2 ≤ 5	>5	16	73	50	52
>5 ≤ 10	>5	55	85	–^a^	–^a^
>10	>5	86	90	86	71

^a^Insufficient number of patients to estimate

^b^Per 50 Fields

## Conclusion

Parietal recurrence of GIST after laparoscopy is rarely reported. They are considered as metastasis. This complication could be avoided with preventive measures such as the use of extraction bags or wound protectors.

## Consent

The patient gave signed statement, which authorises the use of her personal and/or medical information in the publication of this study.

## Abbreviations

GIST: gastrointestinal stromal tumor
CT: computed tomography
SSI: surgical site infection
